# Case Report: Disseminated primary pulmonary carcinoma presenting as a chronic enteropathy in a dog

**DOI:** 10.3389/fvets.2025.1647578

**Published:** 2026-01-30

**Authors:** Yuvani Bandara, Sarah B. Shropshire, Kelly Hughes, Adam Harris, Jenna Hart Burton

**Affiliations:** Colorado State University, Fort Collins, CO, United States

**Keywords:** lipogranulomatous lymphangitis, carcinoma, thyroid transcription factor (TTF-1), duodenum, protein-losing enteropathy (PLE), neoplasia, peritoneal effusion, intestinal thickness

## Abstract

A 10-year-old, male castrated, mixed-breed dog presented with a four-month history of enteropathy unresponsive to standard treatment. Initial bloodwork ruled out non-gastrointestinal illness and abdominal ultrasound revealed stratified segmental small intestinal muscularis thickening. Gastrointestinal endoscopy and biopsy identified duodenal carcinoma with lymphatic infiltration. Due to the absence of an identified gross lesion, nascent primary duodenal carcinoma was suspected. The dog presented 29 days later for acute worsening of signs, development of neoplastic peritoneal effusion, and protein-losing enteropathy. Thoracic radiographs identified pulmonary nodules consistent with carcinoma on cytology. Humane euthanasia was elected 2 days later with necropsy identifying disseminated neoplastic emboli and marked lipogranulomatous lymphangitis. Strong positive nuclear labelling for thyroid transcription factor-1 of the pulmonary neoplasm and neoplastic cells within the small intestinal lymphatics of the original biopsy were consistent with disseminated primary pulmonary carcinoma (PPC). This is the first report describing disseminated PPC presenting primarily with gastrointestinal signs.

## Introduction

Primary pulmonary malignant tumors in dogs comprise epithelial tumors such as adenocarcinoma, carcinoma, and anaplastic carcinoma; and non-epithelial tumors such as sarcomas and neuroendocrine tumors (1, 2). Overall, primary lung tumors in dogs are of low incidence at 1.2% (3–5), with primary pulmonary carcinoma (PPC) accounting for between 87 and 97.1% of these cases (1, 3). Canine PPCs characteristically spread loco-regionally as solid lesions to sentinel lymph nodes, pulmonary parenchyma, and the main bronchi (2, 6). Metastasis beyond this has been reported in 0.9% of cases (1, 6). Isolated case reports describe distant metastasis as gross lesions in the brain, eyes, adrenal glands, and skin (7, 8). Typical clinical signs of disease reflect respiratory abnormalities, namely coughing, dyspnea, hemoptysis, lethargy, and exercise intolerance; however, some dogs can also present with lameness secondary to paraneoplastic hypertrophic osteodystrophy (1, 2, 9–11). Between 5.5 and 30% of cases of primary pulmonary tumors in dogs are found incidentally in the absence of clinical signs (2, 3, 11, 12). This is the first report of a dog diagnosed with disseminated PPC presenting with the sole complaint of a chronic enteropathy, secondary to gastrointestinal metastasis, leading to delayed discovery of the primary tumor.

## Case description

A 10-year-old, male castrated, mixed-breed dog presented to the primary veterinarian (PV) for a three-week history of enteropathy characterized by vomiting, hyporexia, and weight loss. The dog had a prior lifelong history of intermittent gastrointestinal signs which appeared to be food and antibiotic-responsive. At this visit, the dog weighed 38.2 kg and was over-conditioned. A complete blood count (CBC), serum chemistry panel, and total thyroxine all showed no clinically relevant abnormalities. Serum albumin (3.5 g/dL; reference interval [RI], 3.0–4.3 g/dL) and globulin (2.9 g/dL; RI 1.5–3.2 g/dL) were within the reference ranges. Voided urinalysis identified isosthenuria at 1.009, 1 + proteinuria and pH 8.5. A urine protein creatinine ratio measured 0.5 (RI 0.2–0.5). The dog was treated as an outpatient with 2 days of maropitant (2 mg/kg per os [PO] every 24 h) and 5 days of sucralfate (1 gram PO every 12 h) and advised a home-prepared diet of chicken and rice. The dog re-presented to the PV 16 days later for vomiting, hyporexia, and continued weight loss despite symptomatic treatment. Further diagnostics at this visit were declined by the owner, and the dog was prescribed 1 week of both famotidine (1 mg/kg PO every 12 h) and gabapentin (10 mg/kg PO every 12 h). The dog re-presented to the PV again 7 days later for continued clinical signs. An abdominal ultrasound was performed by a board-certified veterinary radiologist. This identified diffuse moderate to marked gastric mural thickening, mild pancreaticoduodenal lymphadenopathy, mild peri-gastric steatitis, scant volume peritoneal fluid, and mild thickening of the cecal wall. The dog was subsequently commenced on a tapering dose of prednisolone (0.5 mg/kg PO every 24 h for 7 days, then 0.25 mg/kg PO every 24 h for 7 days, then 0.25 mg/kg PO every 24 h every other day for 7 days), omeprazole (1 mg/kg PO every 12 h), and maropitant (2 mg/kg PO every 24 h). Following this visit, the dog exhibited an increased appetite, polyuria and polydipsia (attributed to glucocorticoid use), and reduced vomiting frequency. Four weeks after the end of steroid therapy, the dog presented to an emergency veterinarian for acute vomiting and anorexia. The dog was prescribed subcutaneous fluids of lactated ringer’s solution at 25 mL/kg, capromorelin (3 mg/kg PO every 8 h), and ondansetron (0.5 mg/kg PO every 12 h), and recommenced on prednisolone (0.6 mg/kg PO every 24 h). Bloodwork revealed low serum cobalamin (358 ng/L; RI 284–836 ng/L; Texas A&M Gastrointestinal Panel, College Station, Texas), and subcutaneous cobalamin supplementation of 1000mcg weekly for 4 weeks was prescribed.

The dog was referred to a veterinary teaching hospital (VTH) 4 days later for gastrointestinal endoscopy, 4 months since the initial appearance of gastrointestinal signs. On presentation, the dog was of appropriate body and muscle condition for his weight of 35.2 kg, despite a history of chronic weight loss (13). At this time, the dog’s diet consisted of the same home-prepared diet which was estimated to be 10% below the dog’s daily energy requirements based on nutritional calculation of the recommended energy requirement. Physical examination identified normal vital parameters, moderate periodontal disease, and a 1.5-cm circumferential, soft, moveable subcutaneous mass on the right lateral neck. Fine needle aspiration of the mass identified mild mixed inflammation, and no overtly neoplastic cells were noted. Bloodwork was not performed at this visit. Abdominal ultrasound was performed by a board-certified veterinary radiologist. This identified normal gastric wall layering and thickness; increased overall wall thickness of the duodenum and jejunum with mild to moderate muscularis layer thickening in these segments; a diffusely heterogenous liver with multiple variably sized hypo- and hyperechoic nodules with the largest nodule measuring 2.7 cm in diameter; a small 0.27 cm hyperechoic left adrenal gland nodule considered to represent an adenoma or adenomatous hyperplasia; scant peritoneal effusion and a right caudoventral abdominal subcutaneous lipoma. The liver nodules were not sampled due to prioritization of non-neoplastic processes of vacuolar hepatopathy and nodular regeneration based on imaging description and the dog’s history of glucocorticoid administration.

Gastroduodenoscopy and ileocolonoscopy were performed the following day. During the procedure, areas of pinpoint mucosal hemorrhage were noted in the stomach along with moderate villous blunting and yellow granularity in the duodenum, and mild mucosal irregularity of the ileum. The colon appeared grossly normal. At least eight partial-thickness biopsies were obtained from each site. The dog was continued on prednisolone (0.6 mg/kg PO every 24 h) and initiated on a hydrolyzed diet (Nestlé Purina, HA Vegetarian Dry) pending results of histopathology.

**Figure 1:**
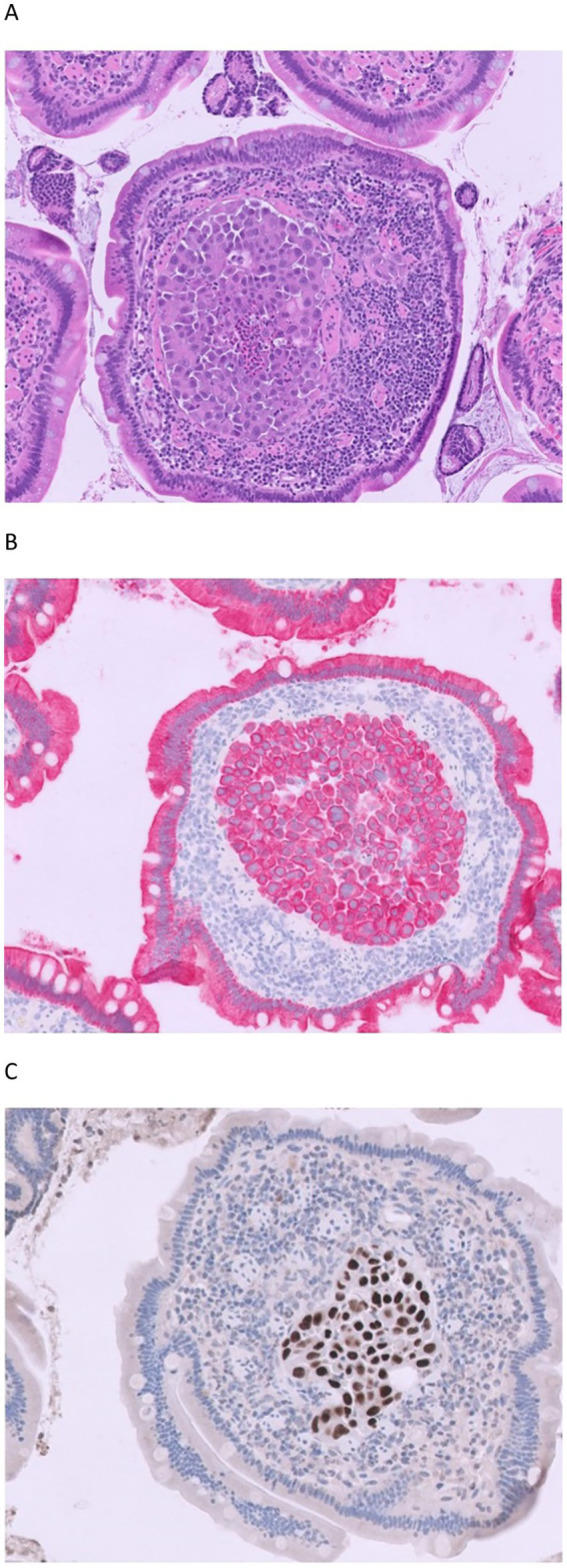
**(A–C)** Duodenal biopsy from a 10-year-old, male castrated, mixed-breed dog presenting with a four-month history of chronic enteropathy characterized by vomiting, hyporexia, and weight loss. There are aggregates of neoplastic epithelial cells within villous lacteals [hematoxylin and eosin (HE) stain, magnification 100×, **(A)**]. Neoplastic epithelial cells have strong cytoplasmic immunoreactivity to cytokeratin (**B**, immunohistochemistry for cytokeratin, 200 × magnification) and strong nuclear immunoreactivity to thyroid transcription factor 1 (TTF-1) (**C**, immunohistochemistry for TTF-1, 200 × magnification).

Initial histopathology of the gastrointestinal biopsy specimens identified mild chronic lymphoplasmacytic gastritis, enteritis (ileum), and colitis. In the duodenal biopsy, which was superficial and primarily consisted of villous tips, neoplastic epithelial cells were found within the villous lacteals (Figure 1A), along with chronic lymphoplasmacytic and eosinophilic enteritis with villous blunting, crypt ectasia, and mild fibrosis. Due to the absence of a gross lesion noted in the duodenum, and peculiarity of identified neoplastic epithelial cells here, cytokeratin immunohistochemistry was performed (Figure 1B). This confirmed neoplasia of epithelial cell origin which was interpreted as duodenal carcinoma.

### Follow-up 1

The dog was followed up 12 days later at the VTH by which time full results from histopathology had returned. The dog had no vomiting but had intermittent hyporexia and continued weight loss. The owner described the dog as not wanting to eat the previously prescribed hydrolyzed diet. On physical examination, vital parameters were normal; however, the dog had lost 1.4 kg since the time of endoscopy now weighing 33.8 kg. A CBC was unremarkable apart from a mild lymphopenia (0.1 K/uL; RI, 1–4.8 K/uL) consistent with a stress leukogram. Serum chemistry identified a mild increase in alanine aminotransferase (198 IU/L; RI, 10–90 IU/L), lower end of normal cholesterol (157 mg/dL; RI, 130–300 mg/dL), hypoalbuminemia (2.4 g/dL; RI 3.0–4.3), and associated mild total hypocalcemia (8.9 mg/dL; RI, 9.0–11.5 mg/dL), lower end of normal globulins (1.8 g/dL; RI, 1.5–3.2 g/dL), and mild total hypomagnesemia (1.8 mg/dL; RI, 1.8–2.4 mg/dL), consistent with a protein-losing enteropathy (PLE). Further diagnostics at this visit were declined and treatment with maropitant (2 mg/kg PO every 24 h), gabapentin (10 mg/kg PO every 8 h), and a different reduced-fat diet (Mars Royal Canin^®^, Canine Gastrointestinal Low Fat Loaf) was elected. The prednisolone was tapered and stopped over 4 weeks (0.3 mg/kg PO every 24 h for 2 weeks, then 0.15 mg/kg PO every 24 h for 2 weeks). As no gross lesion in the duodenum was identified at the most recent abdominal ultrasound, the working diagnosis was discovery of nascent primary gastrointestinal carcinoma. The owner was advised to re-present the dog in 3 weeks’ time for investigation of a developing duodenal lesion and tumor staging with a view for surgical resection.

### Follow-up 2

**Figure 2:**
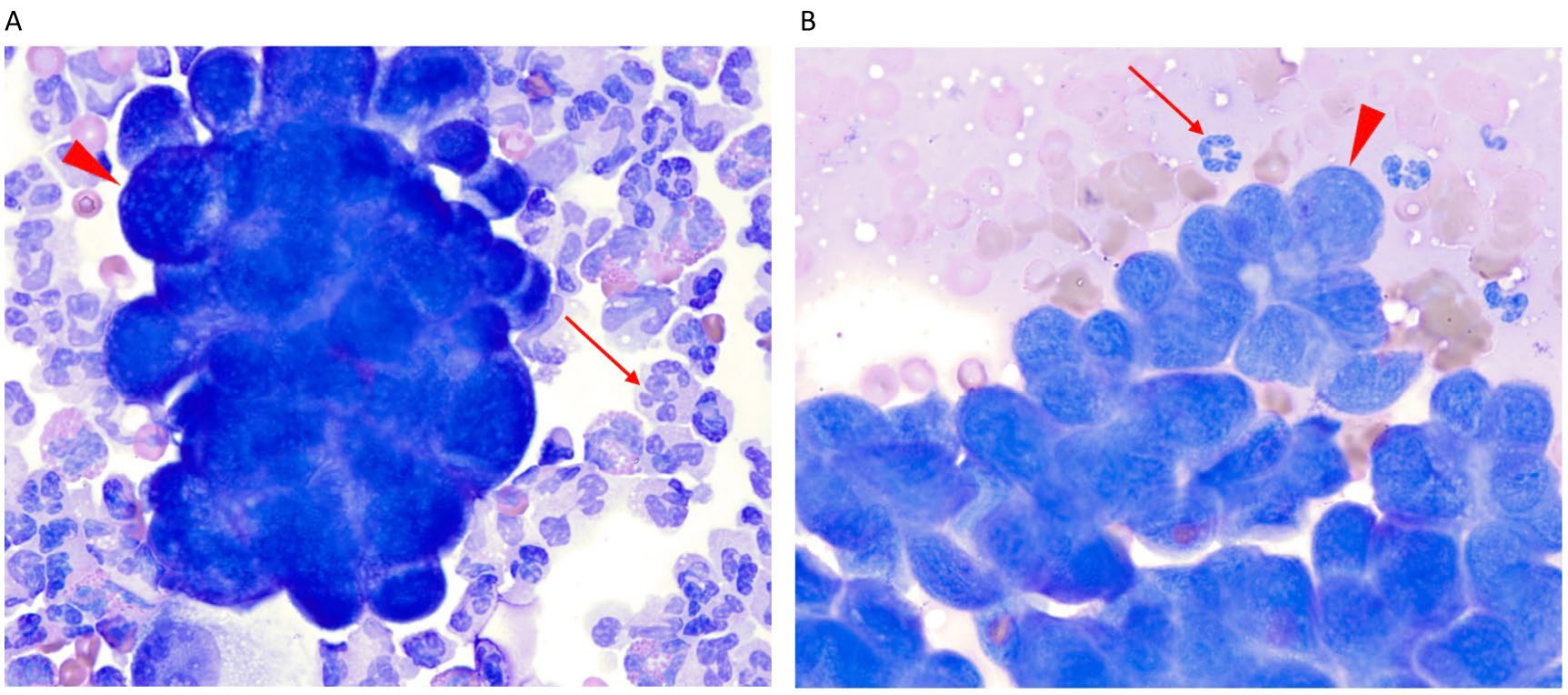
**(A,B)** Representative cytologic features from the same dog as Figure 1, the peritoneal effusion **(A)** and lung mass **(B)**. Red arrows indicate non-degenerate neutrophils, while red arrowheads highlight variably sized clusters of neoplastic epithelial (carcinoma) cells. Both images are stained with Modified Wright-Giemsa, 1,000 × magnification.

The dog re-presented to the VTH 17 days later for acute worsening of signs described as anorexia, weight loss, and weakness. The dog was found to have lost a further 1.7 kg since the last visit, now weighing 32.1 kg and being of notably reduced body condition. Physical examination identified an elevated rectal temperature of 39.7 °C but was otherwise static from previous visits. A CBC showed no clinically relevant abnormalities. Serum chemistry noted increases in creatinine kinase (487 IU/L; RI 50–275 IU/L) and aspartate aminotransferase (58; RI 15–45) suspected to be secondary to hyperthermia, catabolic state, or restraint from blood sampling. Serum cholesterol (130 mg/dL), total magnesium (1.7 mg/dL), and albumin (2.0 g/dL) had all worsened. Voided urinalysis revealed isosthenuria at 1.012, pH 7, and no proteinuria. Thoracic radiographs identified multiple pulmonary nodules and masses coalescing in the right middle and right caudal lung lobes and small volume pleural effusion. Ultrasound-guided fine-needle aspiration of part of the coalescing mass in the right lung lobes identified atypical and tightly cohesive epithelial cells consistent with a carcinoma. Abdominal ultrasound was repeated by a board-certified radiologist with pertinent findings of acute pancreatitis with a moderately hypoechoic and lobulated right pancreatic limb; static left adrenal nodule; moderate hypoechoic thickening (0.38 cm) of the muscularis at the mesenteric border of the proximal descending duodenum; similar to prior mild to moderate muscularis layer thickening of the duodenum and jejunum; diffuse submucosal thickening of the ascending colon, cecum and ileum; and mild peritoneal effusion. No loss of intestinal wall layering was noted. Fluid analysis of the peritoneal effusion revealed a total protein of 1 g/dL, and total nucleated cell count of 1,400/uL. Cytologic evaluation (Figure 2A) identified nucleated cells consisting of 81% neutrophils, 8% eosinophils, 6% lymphocytes, and 5% large mononuclear cells. Clusters of tightly cohesive cells with a high nuclear to cytoplasmic ratio and a scant amount of deeply basophilic cytoplasm were present with cytological resemblance to the sampled pulmonary nodule (Figure 2B), consistent with a neoplastic effusion. Fluid culture yielded no aerobic or anaerobic growth. Due to the patient’s guarded prognosis, the owner elected discharge with no further treatment. The dog was humanely euthanized 2 days later, and necropsy was performed at the VTH diagnostic laboratory by a board-certified veterinary pathologist (KLH).

### Necropsy findings

**Figure 3:**
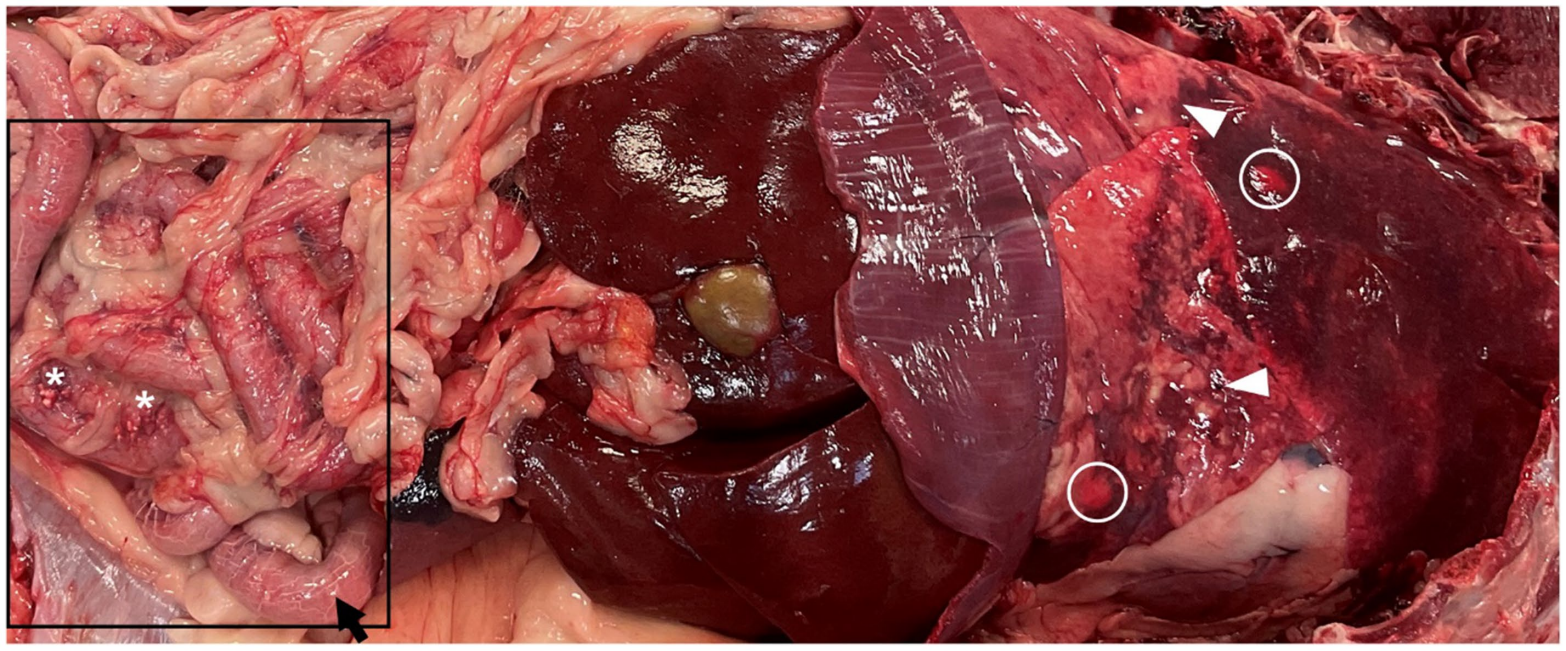
Necropsy from the same dog as Figure 1, approximately 30 days later following the initial biopsy for progressive anorexia, weight loss, and weakness. The lung parenchyma is irregular and firm with multifocal tan to white discrete (circles) to coalescing nodules (arrowheads). The small intestines are within the box. Along the mesenteric surface of the small intestine, there are numerous white, firm nodules (*) with dilated serosal lymphatics (arrow).

**Figure 4:**
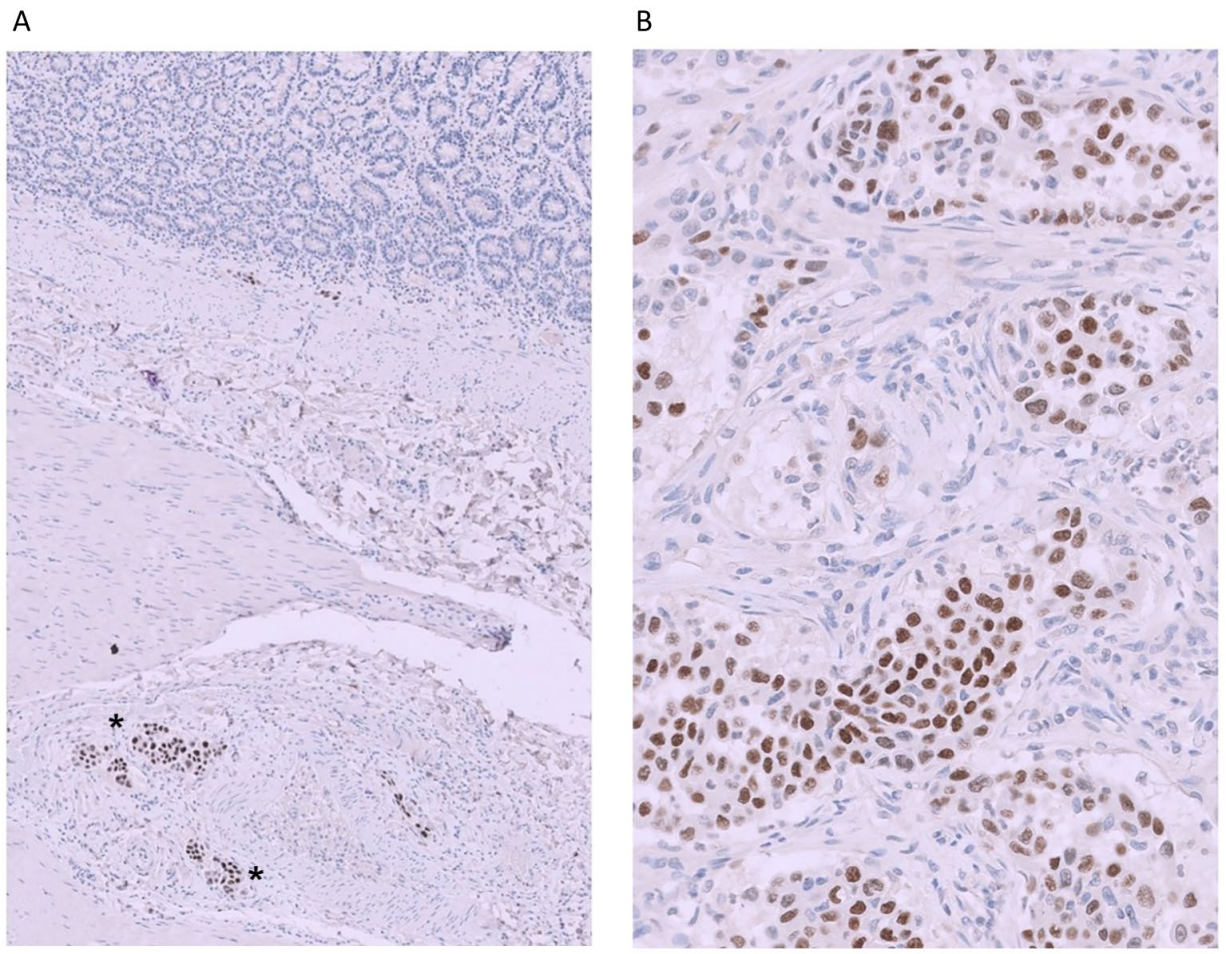
**(A,B)** The lung parenchyma is effaced by poorly differentiated neoplastic epithelial cells either forming papillary structures (**A**, left side) supported by dense fibrous stroma with intermixed necrosis (*) or forming micropapillary structures with central aggregates of neoplastic cells (**A**, right side, HE, 50 × magnification). The neoplastic cells in the lung have nuclear immunoreactivity to TTF-1 (**B**, immunohistochemistry for TTF-1, 100 × magnification) as seen in the small intestine (Figure 5B), liver, heart, and kidney (not shown) and the original duodenal biopsy (Figure 1C).

**Figure 5:**
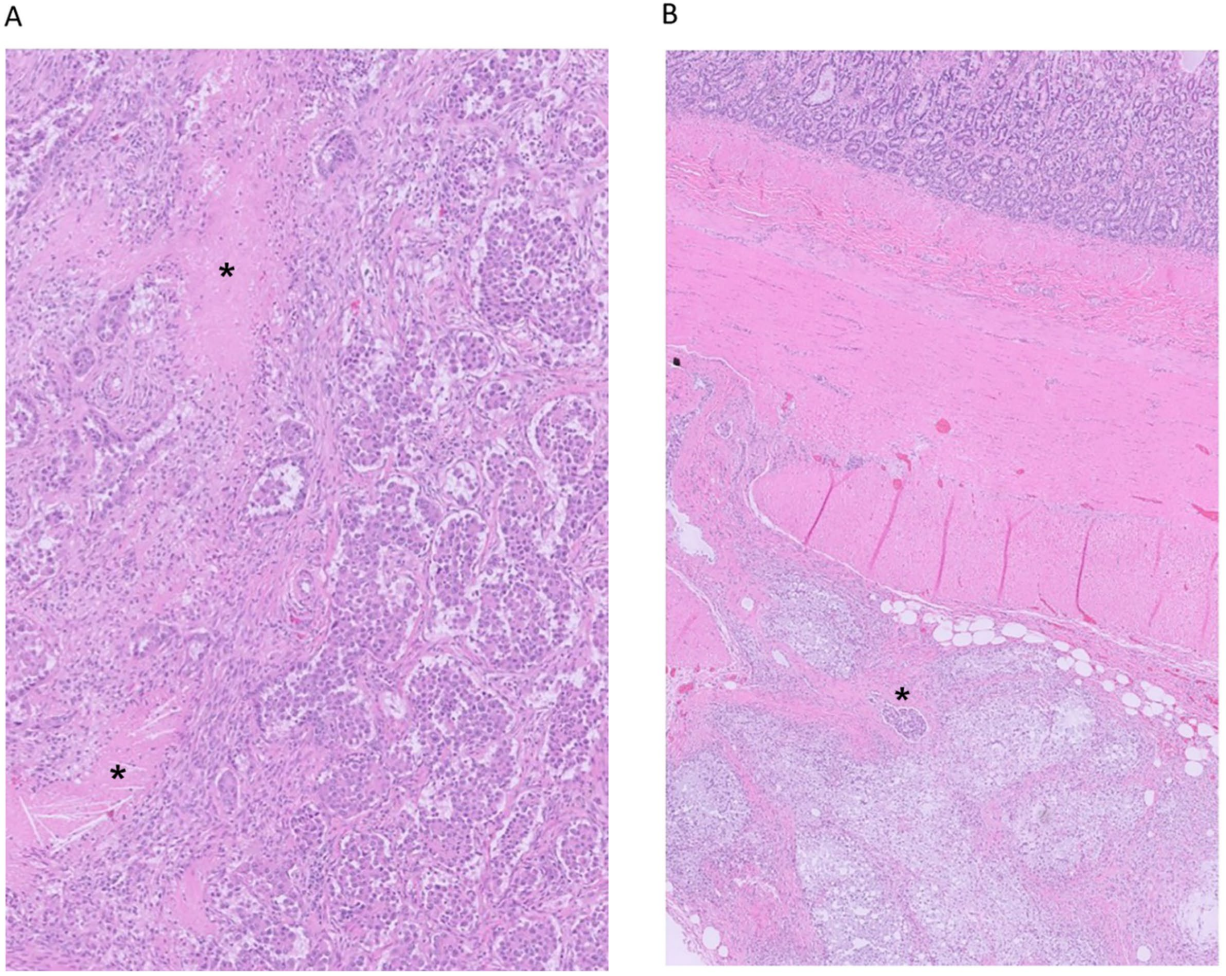
**(A,B)** Small intestine from necropsy of a 10-year-old mixed-breed dog. The serosa is expanded by lipogranulomatous aggregates associated with lymphatic leakage (A, bottom, HE, 20 × magnification). There are transmural lymphatics with neoplastic emboli composed of neoplastic polygonal cells (**A**, *, HE, 20 × magnification) with nuclear expression of TTF-1 (**B**, *, immunohistochemistry for TTF-1, 50 × magnification).

Gross necropsy examination identified multifocal to coalescing, firm, tan to white nodules affecting approximately 50% of the pulmonary parenchyma, notably the caudal lobes (Figure 3). Upon dissection, the nodules had roughened, mottled, tan to dark-red surfaces. In the intestinal tract, there were numerous white, firm nodules along the mesenteric surface with dilated and prominent serosal lymphatics most consistent with (lipo)granulomatous lymphangitis (Figure 3, square). In addition, there was a mild amount (~50 mL) of chylous effusion in the abdominal cavity. No overt mass effect was identified within the duodenum or other gastrointestinal segments during gross or microscopic evaluation. Standard tissues were collected and fixed in 10% neutral buffered formalin, processed routinely, and embedded in paraffin wax. Sections at 5 μm thickness were stained routinely with hematoxylin and eosin (HE). Sections of the lung, kidney, spleen, thyroid, heart, small intestines, and colon were examined microscopically. Histopathologic examination of HE-stained sections of the lung revealed multifocal to coalescing accumulations of poorly differentiated neoplastic epithelial cells effacing and replacing normal parenchyma. Neoplastic cells occasionally showed a more papillary growth pattern, with branching growth into alveolar spaces, often with abundant subepithelial fibrous stroma with many regions characterized by micropapillary or solid growth with solid accumulation of pleomorphic neoplastic cells (Figure 4A). These multiple patterns classified the tumor as having a mixed pattern. Neoplastic cells were columnar to polygonal with moderate to abundant amounts of eosinophilic cytoplasm and distinct cell borders. Nuclei were round to irregularly round with finely stippled to vesicular chromatin and occasionally a prominent nucleolus. Some neoplastic cells had a centralized vacuole with eccentrically displaced nuclei (signet ring cell). There were nine mitotic figures per 10 high-power fields. (2.37mm^2^). Neoplastic cells were frequently found partially occluding vessel lumens throughout the remaining parenchyma. Due to these characteristics, immunohistochemistry with thyroid transcription factor-1 ([TTF-1], clone 8G7G3/1, Dako, mouse monoclonal, 1:150 dilution) was performed on the lung, kidney, liver, heart, and small intestine which identified strong positive nuclear labelling in the lung (Figure 4B) and small intestine (Figure 5B). This confirmed a diagnosis of PPC in the absence of a thyroid tumor (14). In light of this finding, TTF-1 immunohistochemistry was performed on the original duodenal biopsy which showed strong nuclear immunoreactivity of the neoplastic cells within lacteals (Figure 1C). Small neoplastic emboli were frequently identified within the lumen of vessels within the heart, liver, kidneys, small intestine, and colon. Sections of HE-stained small intestine found numerous lymphatic vessels throughout the intestinal wall variably occluded by neoplastic cells of similar histomorphology as described within the lungs (Figures 5A,B). Additionally, there was a marked lipogranulomatous lymphangitis (LL) within the deep muscular layers and along the serosa (Figure 5A) consistent with gross findings (Figure 3). Crypt abscesses were noted and lacteals moderately ectatic ranging in size from 60 to 70% of the width.

## Discussion

This report describes a case of disseminated PPC with metastasis of neoplastic vascular and lymphatic emboli to the heart, kidneys, liver, and gastrointestinal tract. No respiratory signs, as expected with pulmonary neoplasia, were noted throughout the dog’s multiple clinical evaluations despite neoplasia affecting approximately 50% of the pulmonary parenchyma on necropsy. The absence of these signs consequently led to a delayed diagnosis of PPC due to focus on an emerging primary duodenal carcinoma. The use of TTF-1 immunolabelling allowed for confirmation of PPC and has been associated with a poorer prognosis in human pulmonary carcinoma across all disease stages (14–16). However, the prognostic significance of TTF-1 expression in dogs has not been determined. The hyperechoic liver on ultrasound was considered most consistent with vacuolar hepatopathy and nodular regeneration, especially given the dog’s history of glucocorticoids and was therefore not sampled during the initial workup. It is plausible that liver aspirates could have led to an earlier diagnosis of neoplasia, but given the lack of true tissue infiltration and rare vascular invasion noted on histology, liver aspiration could have easily missed neoplastic cells. Our case highlights the reality of diagnostic reasoning being guided by the most plausible interpretation of findings available to the clinician at that time. This simultaneously demonstrates that atypical architectural changes may not align with clinical expectations and may still justify further investigation.

As the dog’s presenting signs were most compatible with a chronic enteropathy with clinical progression into a PLE, this led to a focused gastrointestinal workup. The PLE most plausibly occurred due to progressive LL, secondary to neoplastic embolization of lymphatics observed on duodenal histopathology at the time of necropsy. Lipogranulomatous lymphangitis is thought to arise secondary to inflammation triggered by chronic, localized leakage of lipid-rich chyle from compromised or blocked lymphatic vessels (17, 18). This process typically results in marked intestinal wall thickening, stenosis, or the formation of gross granulomatous lesions. A multifactorial etiology, comprising infectious, genetic, and inflammatory causes, has been proposed (19). However, many cases can be idiopathic. Lipogranulomatous lymphangitis can also theoretically occur secondary to lymphatic obstruction from solid neoplasia; however, this is yet to be reported. Our case highlights the inclusion of neoplasia as a differential diagnosis in dogs with histologic evidence of LL despite macroscopic changes.

Intestinal tumors are rare in dogs with a reported incidence of 1.9–8%, presenting mostly in dogs over 6 years of age (20–22). Common canine intestinal neoplasms are carcinoma, lymphoma, mesenchymal tumors (leiomyomas, leiomyosarcomas), and stromal tumors (23). Adenocarcinoma represents the most common malignant intestinal neoplasm in dogs predominantly affecting the colon and rectum (24, 25). Small intestinal carcinomas characteristically develop as solitary masses with at least 48% of these being detected on initial abdominal radiographs or ultrasound (23, 26–31). Typical ultrasonographic changes are focal intestinal wall thickening, complete loss of wall layering, or an overt mass effect. Loss of wall layering is found in 99% of dogs with intestinal neoplasia in contrast to the preservation of wall layering in 88% of dogs with non-neoplastic disease (5). This dog showed stratified intestinal wall thickening on three ultrasound examinations which precluded an earlier diagnosis of neoplasia.

The feature of a solitary intestinal mass dictates the mainstay of therapy which is wide tumor resection and local lymph node extirpation. Limited information is present regarding the prognosis of dogs with small intestinal carcinoma without treatment; however, surgery, with or without adjuvant chemotherapy, carries a survival rate of 60% after 1 year (29, 32). With this in mind, we felt it was rational to closely monitor the dog for the development of a measurable gross lesion before proceeding with surgical intervention. Additionally, despite thoracic imaging typically being part of routine staging in cancer patients, this was planned to be performed at the time of evaluation of a gross lesion. Previous reports have identified that metastasis to the lungs is uncommon in dogs with non-lymphomatous intestinal neoplasia (27, 29, 32).

Gastrointestinal metastasis of malignant pulmonary neoplasms in humans is rare and has a reported incidence of 1.77% (33). In humans, the subtypes of large cell carcinoma, small cell pulmonary neoplasia, and squamous cell carcinoma have demonstrated a higher risk of metastasizing to the gastrointestinal tract, whereas adenocarcinomas exhibit a lower risk (34). In human patients with a known history of primary pulmonary neoplasia, the development of gastrointestinal symptoms, usually due to intestinal hemorrhage, perforation, and/or obstruction, is a feature of end-stage metastatic disease (34–36). Pulmonary neoplasia is considered a prioritized differential for unexpected bowel perforation in heavy smokers over the age of 50 (37, 38). Urban living and second-hand smoke exposure have both been associated with primary lung cancers in dogs, but a true causal relationship has not been clearly established (39, 40). These risk factors were not present for this dog, and this information was only gathered from the owner following the dog being euthanized. Further information is still required to evaluate the impact of environmental exposures on the development of primary pulmonary neoplasia in dogs.

Retrospective evaluation of our case highlights the consideration of searching for a distant primary tumor when presented with an older dog with histopathologic confirmation of intestinal carcinoma in the absence of a typically expected gross lesion. This underscores that unexpected presentations of neoplasia should prompt thorough evaluation of both tumor and metastatic behaviors to inform a targeted diagnostic workup. This case is an unusual example of PPC with distant metastasis. Carcinomas typically metastasize through lymphatics which can progress to vascular spread later in the disease course as was identified in this case. Due to the rich blood supply to the gastrointestinal tract, this organ should be considered a site for metastasis even with atypical cancer types. Neoplasias in people that have been described to commonly metastasize to the gastrointestinal tract are cancers of the breast, ovary, urogenital system, and melanomas (41). Similar relationships are yet to be conclusively identified in veterinary patients. Thorough cancer screening should be of consideration in dogs over the age of eight during diagnostic workup of chronic and progressive clinical signs (42).

## Data Availability

The raw data supporting the conclusions of this article will be made available by the authors, without undue reservation.
